# Monolayer/Bilayer Equilibrium of Phospholipids in Gel or Liquid States: Interfacial Adsorption via Monomer or Liposome Diffusion?

**DOI:** 10.3390/gels9100803

**Published:** 2023-10-06

**Authors:** Kirsten Ullmann, Lea Fachet, Hermann Nirschl, Gero Leneweit

**Affiliations:** 1 Karlsruhe Institute of Technology (KIT), Department of Mechanical Process Engineering and Mechanics, Straße am Forum 8, 76131 Karlsruhe, Germany; hermann.nirschl@kit.edu; 2 Independent Researcher, 76131 Karlsruhe, Germany; 3 Carl Gustav Carus-Institute, Association for the Promotion of Cancer Therapy, Allmendstr. 55, 75223 Niefern-Oeschelbronn, Germany

**Keywords:** phospholipids, monomer and liposomal diffusion, perfluorocarbon, interfacial tension, tensiometry, temperature

## Abstract

Phospholipids (PLs) are widely used in the pharma industry and a better understanding of their behavior under different conditions is helpful for applications such as their use as medical transporters. The transition temperature T_m_ affects the lipid conformation and the interfacial tension between **p**er**f**luoroperhydro**p**henanthrene (PFP) and an aqueous suspension of 1,2-dipalmitoyl-sn-glycero-3-phosphatidylcholine (DPPC), 1,2-distearoyl-sn-glycero-3-phosphatidylcholine (DSPC), as well as a mixture of these PLs with cholesterol. Interfacial tensions were measured with the Du Noüy ring at quasi-equilibrium; the area per molecule was calculated according to the Gibbsian approach and a time-dependent tension gradient. Results show that the time *t_ε_* to reach quasi-equilibrium was shorter when the temperature was above T_m_, indicating a faster adsorption process (*t_ε_*_,DPPC_,_36 °C_ = 48 h, *t_ε_*_,DPPC,48 °C_ = 24 h) for PL in the liquid crystalline state than in the gel state (T < T_m_). In addition, concentration-dependent results of the interfacial tension revealed that above the respective T_m_ and at all concentrations *c* > 0.1 mM, the average minimum interfacial tension for DPPC and DSPC (14.1 mN/m and 15.3 mN/m) does not differ significantly between those two lipids. Equilibrium between monolayers and bilayers shows that for T < T_m_, surface pressures *∏* ≈ 31 mN/m are reached while for T > T_m_, *∏* ≈ 41 mN/m. Mixtures with cholesterol only reach *∏* ≤ 31 mN/m T_m_, with no significant difference between the two PLs. The higher interfacial tension of the mixture indicates stabilization of the liposomal conformation in the aqueous phase by the addition of cholesterol. The high diffusion coefficients show that adsorption is mainly based on liposomes.

## 1. Introduction

Phospholipids (PLs) are abundantly used in the pharmaceutical and food industry as natural emulsifiers [1,2]. The invention of efficient loading technologies of drugs into liposomes by remote loading and phase transfer of the drug into a gel allowed the development of a drug delivery system capable of achieving accumulation in, e.g., tumours and reducing side effects [3,4]. The development of mRNA as potential active pharmaceutical ingredients started with the use of PLs as a delivery agent forming liposomes [5]. However, difficulties of low encapsulation rates led to the invention of cationic lipids [6]. The discovery of the toxicity of cationic lipids in the human body initiated the synthesis of ionizable lipids [7,8]. These inventions enabled the development of novel vaccination concepts in recent years based on DNA, siRNA, and mRNA transfection [9]. However, such lipid nanoparticles formed by ionizable lipids still suffer from a very inefficient endosomal escape of only 1–2% [10]. These deficits kindled the recent elaboration of novel synthetic PLs that enable organ-selective mRNA delivery and CRISPR–Cas gene editing [11]. The versatility in the design of lipids containing both hydrocarbon chains and phosphorous groups for pharmaceutical use by either natural or synthetic PLs proves the flexibility of these compounds and their physiological relevance.

This adaptability of PLs also enables different modes of formation of structural colloidal elements in condensed gel or in different liquid states such as bilayer lamellar phases, monolayers, and reverse and regular micelles, as well as hexagonal H_II_ phases. This variety is known as lipid polymorphism and enables dynamic changes within milliseconds as can be studied in, e.g., synaptic vesicles [12]. Another field of physiological relevance is the formation of neutral lipid droplets (LDs) in the endoplasmic reticulum (ER), covered by a PL monolayer. Biogenesis of LDs is enabled by the supply of the ER’s PL bilayer, allowing the terminal budding of an LD from a cell organelle’s membrane. During LD formation, thermodynamic equilibrium between a PL monolayer and bilayer phase exists [13]. It is the close similarity of PL monolayers and bilayers to physiological processes that offers insights into complex biophysics. Moreover, a deeper understanding of the biophysics of PL monolayers and bilayers has triggered many recent developments in tomorrow’s targeted drug delivery systems based on PLs [14,15].

The amphiphilic characteristics of PLs allow the encapsulation of both lipophilic and hydrophilic drugs, either within the lipophilic area of the bilayer membrane or in the aqueous core of the vesicle. In addition, the shape of the PLs (e.g., conic or cylindric shape) influences the shape of the bilayer—a conic PL forms a vesicle of high curvature while a cylindrical shape is in an energetically lower state in a planar bilayer conformation of low curvature [16]. Furthermore, interfacial behavior, shape, stability, emulsification and encapsulation efficacy are influenced by environmental parameters such as osmotic conditions or temperature. The latter is discussed in this work by virtue of its fundamental influence on the mobility, emulsification and drug encapsulation potential of PLs.

Each PL has a so-called main transition temperature T_m_ at which its state changes. Below this temperature, PL monolayers or bilayers appear in a gel-like and poorly mobile state, while above this temperature they gain mobility and attain a more fluid constitution. The increased fluidity also changes the adsorption or desorption process to the interface by interpenetration into an existing mono- or bilayer. In principle, PLs with long, saturated fatty acid chains have a higher transition temperature than short-chain or unsaturated PLs [17]. In addition to the chain length, the degree of saturation of the fatty acids, the head group and the purity of the lipids also play a role. Thus, especially for natural PLs comprising different lipids in complex mixtures, the transition temperature is not abrupt, but instead occurs over a temperature range [18,19].

There are numerous temperature studies on surfactants. Kučerka et al. studied the fluid phase areas and bilayer thickness of different PLs by using small angle X-ray scattering [20]. They found out that with higher temperatures the bilayer thickness decreases. Simultaneously, the trans-gauche isomerization of fatty acids increases with an increase in temperature. Similar observations regarding the phase transition of PLs were published by Leonenko et al. [21]. One of the first studies of temperature and surface/interfacial tension was performed by Purdon et al., who examined the effect of temperature on the surface tension and critical micelle concentration (CMC) of egg lysolecithin [22]. The CMC becomes greater with an increase in temperature while the surface tensions at concentrations below the CMC undergo a minimum; above the CMC, a monotonous minimization occurs. Similar findings were reported by Ye et al., who investigated the change of interfacial tension between crude oil and a gemini surfactant [23]. Other authors examined the interfacial dependence of PLs [24,25].

For emulsification procedures, perfluorocarbons (PFC) came to the fore in recent years. PFCs are biocompatible, inert and stable and therefore of high interest for biomedical emulsions [26,27,28]. For example, PFCs are applied as contrast agents for magnetic resonance imaging (MRI) or are part of compositions for protein delivery [29,30]. Besides their increasing practical use, they are also a suitable model system for biophysical studies due to their omniphobic nature with minimal interactions with PLs. However, there are only very few publications on the interfacial influence of natural PLs and PFC oils [24,31]. In addition, the temperature dependence of PLs in the PFC **p**er**f**luoroperhydro**p**henanthrene (PFP) has not been examined yet. Due to the temperature influence on the conformation of PLs, it is necessary to examine the influence on the interfacial tension between PFP and natural surfactants in order to support proper emulsification for further applications. 

This work examines the temperature dependence of the widely used PLs 1,2-dipalmitoyl-sn-glycero-3-phosphatidylcholine (DPPC) and 1,2-distearoyl-sn-glycero-3-phosphatidylcholine (DSPC), as well as their mixtures with cholesterol. It examines the hypothesis that: (1) the minimal interfacial tension above the transition temperature does not differ regardless of the PL chain lengths; (2) the adsorption process is determined by liposome diffusion; and (3) the state of the monolayer phase is decisive for PL interpenetration. The experimental results are discussed and interpreted by a comparison to recent theoretical and experimental literature.

## 2. Results and Discussion

### 2.1. Temperature Dependence of DPPC and DSPC and Cholesterol

**Figure 1:**
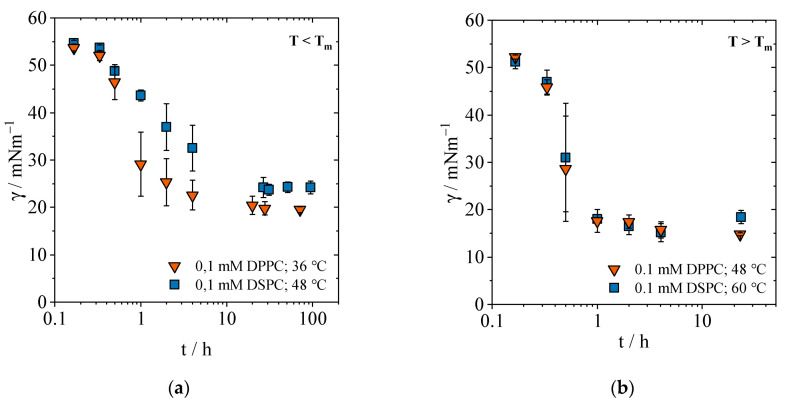
Interfacial tension *γ* as a function of time *t* in dependence of the temperature for the PLs DPPC and DSPC at a concentration of 0.1 mM. (**a**) Setting of the quasi-equilibrium below the respective transition temperature. (**b**): Setting of the quasi-equilibrium above the respective transition temperature. The different time span of the *x*-axis should be noted.

To effectively measure the temperature effect, a temperature difference of 5 °C below the T_m_ of DPPC and 5 °C above the T_m_ of DSPC was set for the PLs studied. In addition, a direct comparison of both PLs was made at 48 °C (at equidistance from the T_m_ of DPPC and DSPC). Thus, measurement series for both lipids are available below (Figure 1a) and above (Figure 1b) the transition temperature. The mixtures with cholesterol were measured at the same temperatures as the respective pure PL.

#### 2.1.1. Equilibrium Interfacial Tensions

Quasi-equilibrium *γ_ε_* was determined for both lipids and the cholesterol/lipid mixture below and above the transition temperature at a concentration of 0.1 mM. As depicted in Figure 1a, for both lipids the interfacial tension at *t* = 0 h is 55 mN/m, corresponding to the interfacial tension between PFP and pure water. Within the first 10 h, the interfacial tension of both PLs decreases. In comparison, DPPC eventually reaches a lower final value of 19.5 mN/m than DSPC with 24.2 mN/m. The initial pronounced lowering of the interfacial tension by DPPC occurs at low surface coverage and is thus accompanied by the low steric hindrance. In the initial phase, when a small change (or inaccuracy) in surface age still has a large effect on the change in interfacial tension, larger error bars are seen than in the final state. Below the transition temperature, the PLs are still in a gel state and poorly mobile, so that the interpenetration process of additional PL molecules in the monolayer is inhomogeneous. This results in larger differences in the measured interfacial tension and higher standard deviations. Nevertheless, compared to 20 °C, the results show that an increase in the temperature range T < T_m_ leads to a considerably faster adsorption process and *t_ε_* at quasi-equilibrium *γ_ε_* was determined with 24 h and 48 h for DPPC and DSPC, respectively [31].

Raising the temperature above the respective T_m_ (Figure 1b) accelerates the adsorption process. The interfacial tension of both PLs decreases to below 20 mN/m within less than one hour and reaches a minimum value of 14.8 ± 0.33 mN/m (DPPC) and 18.4 ± 1.4 mN/m (DSPC), respectively. Already after one hour, the measured values show a small standard deviation. The standard deviation is derived from the different *γ*(*t*) at each measurement for the same time *t* when each interfacial tension of three independent samples was measured (triplicates were measured consecutively and an average value of triplicates is depicted in Figure 1). That in turn leads to a distinction in the interfacial tension. Hence, the smaller the standard deviation for *γ*, the more advanced and closer to equilibrium is the adsorption process of PLs at the interface. At temperatures above T_m_, the measured interfacial tensions of both PLs coincide. Obviously, in the high mobility state, the chain length has no influence on the interfacial tension. Due to the increased fluidity of the PLs, a faster adsorption at the interface takes place. At the same time, the higher temperature increases the diffusion rate and thus accelerates the adsorption process. Steric hindrance by the fatty acid chains is not visible comparable to T < T_m_, as high mobility is the predominant mechanism. Quasi-equilibrium is reached after 24 h for both PLs; there is no detectable difference for the quasi-equilibrium time above T_m._ Consequently, equilibrium times are much shorter at higher temperatures.

**Figure 2:**
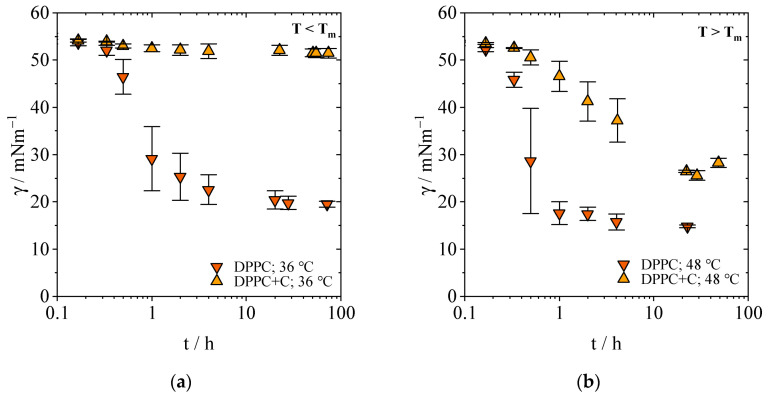
Interfacial tension *γ* as a function of time *t* in dependence of the temperature for the pure PL DPPC and in a mixture with cholesterol (DPPC+C = 60:40 mol-%) at a concentration of 0.1 mM. (**a**) Evolution towards the quasi-equilibrium *γ_ε_* below the transition temperature of DPPC. (**b**): Evolution towards the quasi-equilibrium *γ_ε_* above the transition temperature of DPPC.

Figure 2 depicts the influence of cholesterol on equilibrium time with regard to different temperatures. The mixture of DPPC and cholesterol (DPPC+C) shows that cholesterol reduces the interfacial tension to 51.4 mN/m below T_m_ and to 25.6 mN/m above T_m_ at a concentration of 0.1 mM. For DSPC+C it is 52.3 and 29.6 mN/m, respectively. The molar ratio of 60:40 of PL and cholesterol leads to fewer PLs that are actually available for adsorption and cholesterol does not compensate the smaller quantities to influence the interfacial tension similar to pure PLs. An increase in temperature reduces the interfacial tension further in comparison to pure DPPC. While the difference *Δγ* at the studied temperatures is 4.7 mN/m for DPPC (19.5 mN/m at 36 °C vs. 14.8 mN/m at 48 °C), it is 25.8 mN/m for the mixture DPPC+C (from 51.4 mN/m at 36 °C to 25.6 mN/m at 48 °C). Hence, the temperature effect is more visible for DPPC+C. It can be concluded that the addition of cholesterol stabilizes the liposomes in the stock suspension, impeding the transformation of the liposomal bilayer to the interfacial monolayer. 

The mixture of DSPC+C (graphs available in the Supplementary Material, Figure S1) shows a similar behavior. Below T_m_, interfacial tension does not differ from the interfacial tension between water and the PFP while a temperature increase to 60 °C lowers the interfacial tension to 29.6 mM. The equilibrium time is still long for DSPC+C (168 h and 144 h above T_m_) and does not differ from pure DSPC at 20 °C. In our study, we also detected that the equilibrium time for the mixture is the same as for pure DSPC at 20 °C. Lee et al. found that the addition of cholesterol does not change the equilibrium time nor the interfacial tension [32]. While the first is in accordance with our experiments, the latter is not applicable—apparently due to the differences in the surfaces (water/PFP vs. water/air). At the same concentration but with cholesterol mixed with the PLs, the interfacial tension is much higher than without it. In addition, one would expect to see a difference in equilibrium time and interfacial tension with an increasing temperature. However, while pure DSPC and DPPC adsorb faster at the interface with increasing temperature, this does not occur when cholesterol is added. Cholesterol is known to stabilize membranes making them more flexible, hence leading to fewer breakage of membranes [33]. We can conclude that the stabilizing effect of cholesterol on membranes of liposomes in the aqueous phase is greater than the temperature effect.

A direct comparison of the quasi-equilibrium times t_ε_ at the studied temperatures in Table 1 summarizes the shortening of the adsorption process. For the lipid DPPC, less than 48 h are required at 20 °C until quasi-equilibrium is reached; DSPC, however, requires less than 168 h at 20 °C. An increase to 36 and 48 °C reduces the quasi-equilibrium time while above T_m_, the quasi-equilibrium time can be reduced to 24 h for both lipids. This is due to the more fluid constitution. On the contrary, cholesterol does not influence the quasi equilibrium time. Independent of the temperature, the average time *t_ε_* to reach quasi-equilibrium *γ_ε_* is 48 h for DPPC+C, which is the same as pure DPPC at 20 °C. For the mixture of DSPC+C, a *t_ε_* of 168 h was determined and is only reduced by 24 h at the higher temperature. These quasi-equilibrium times were used for determination of change in interfacial tension depending on concentration.

#### 2.1.2. Concentration-Dependent Measurements

**Figure 3:**
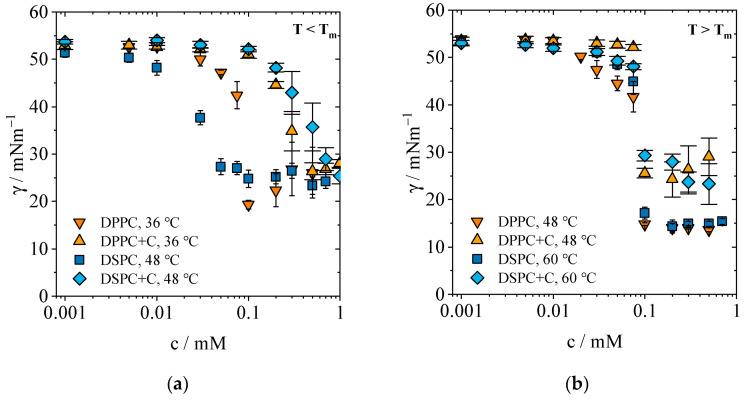
Change in interfacial tension *γ* versus concentration *c* of PLs DPPC, DPPC+C and DSPC at different temperatures. (**a**) Change in interfacial tension below the respective transition temperatures T_m_. (**b**) Change in interfacial tension above the respective T_m_.

To perform concentration-dependent measurements at higher temperatures, the shorter equilibration allowed an acceleration of the experimental procedure for pure PLs. Even at higher temperatures, the initial interfacial tension at very low concentrations *c* < 0.01 mM is within the range of values for pure water and PFP. With increased lipid amount, the interfacial tension decreases as expected. Figure 3a illustrates that DPPC reaches a minimum value of 19.3 mN/m (*c* ≥ 0.1 mM) below T_m_, while the minimum value for DSPC is 24.2 mM (*c* ≥ 0.1 mM). These results are in agreement with the experiments at 20 °C from previous investigations [31]. This means that although the dynamics of adsorption are affected by temperature, the equilibrium interfacial tension is not influenced by T for T < T_m_. At this stage with T < T_m_ and for the PFP interface, *γ_ε_* ≈ 19 mN/m for all *c* ≥ 0.1 mM, resulting in the maximum adsorptive packing density of the monolayers. The increase in interfacial tension at *c* > 0.1 mM could be due to delayed convergence towards equilibrium at higher concentrations. In comparison to Lee et al., who investigated the surface tension of DPPC at the water/air interface at 1 mM, the interfacial tension at the PFP interface is much lower [32]. At the respective temperatures, Lee et al. determined a surface tension of *γ*_surface_ ≈ 40 mN/m for both PLs.

Above T_m_, as shown in Figure 3b, significantly lower equilibrium interfacial tensions are established. The course of both PLs overlaps above the respective T_m_ and shows a strong decrease in the interfacial tension for both phospholipids to γ_ε_ = 13.7 mN/m for DPPC (*c* = 0.5 mM) and to 14.3 mN/m for DSPC (*c* = 0.075 mM), respectively. We hypothesize that the average interfacial tensions $\bar{\gamma }$ of both lipids for *c* > 0.1 mM equal each other and that the chain length no longer has an effect above T_m_, as the increased fluidity and reduced steric hindrance allows lipids to cluster closer together. Thus, the higher PL mobility not only ensures acceleration of the adsorption process, but also a decrease in the minimum interfacial tension. The hypothesis was confirmed with a two-sided t-test for independent samples and with a level of significance α = 0.05. Degrees of freedom (df) for two independent groups was calculated by

(1) $$\mathrm{df}=n_{1\;}+n_{2\;}-\;2$$

where *n*_1_ and *n*_2_ are the number of samples in each group. The values of the *t*-test are summarized in Table 2.

While Lee et al. describe a minimum surface tension of ≈22 mN/m for both PLs above T_m_, which is higher than our results, these authors are also unable to describe a difference between the chain lengths once the temperature rises above T_m_. The results presented here are specific for the interface between PLs and PFP, leading to the conclusion that the lowest possible interfacial tension between PFP/water and PLs does not fall below an average value of *γ_ε_* ≈ 14 mN/m, regardless of the PL.

For the mixture of DPPC and cholesterol, the decrease in interfacial tension starts at a higher concentration than 0.1 mM at T < T_m_. The lowest interfacial tension measured is 26.4 mN/m for both DPPC+C and DSPC+C and contrary to pure PLs, the temperature rise above T_m_ does not lower the minimum interfacial tension significantly (24.3 mN/m). While cholesterol is known to slightly decrease the transition temperature of DSPC-cholesterol mixtures, it moderately influences the interfacial tensions by hindering lipids to adsorb at the interface [34]. For DPPC-cholesterol mixtures, NMR results do not prove a main transition temperature T_m_ for cholesterol fractions >25 mol-% [35]. However, Miyoshi et al. derived a phase diagram where temperature transitions are visible even for cholesterol fractions above 40 mol-% [36]. For the mixture of DSPC+C, the lowest interfacial tension of 25.3 mN/m is reached at a concentration of 1 mM and a slight decrease to 23.3 mN/m is detected above T_m_. Above T_m_, the overall average value at *c* > 0.1 mM is *γ* = 26.2 mN/m at, calculated from all values of both DPPC+C and DSPC+C. A two-sided t-test for independent samples was performed to investigate the hypothesis that the average interfacial tension at *c* > 0.1 mM does not differ between the mixtures of DPPC+C and DSPC+C. The level of significance is α = 0.05 and the degrees of freedom (df) equals 7. The results of the t-test support the hypothesis, leading to the conclusion that indeed in thermodynamic equilibrium between monolayer and liposomes and above the T_m_, the interfacial tension does not change anymore, regardless of the type of PL. *t*-test values can be found in Table 2.

**Figure 4:**
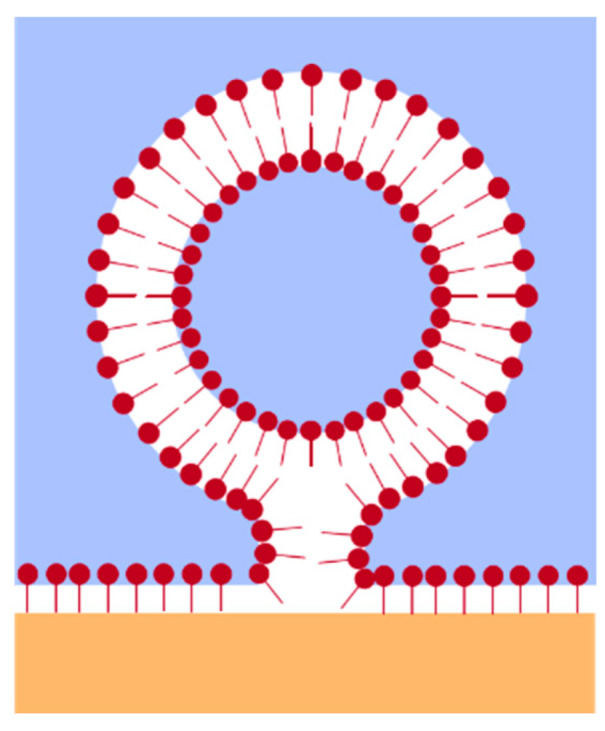
Spreading of PL forming a monolayer at the water/PFP interface, supplied by liposomal bilayers dispersed in the aqueous phase when coalescing with the interface. The geometrical constellation leads to the formation of a thermodynamic equilibrium between the PL monolayer and bilayer conformation, hence referred to as “monolayer–bilayer equilibrium”, MBE (sketch based on [37], modified in simplified form).

The formation of thermodynamic equilibrium between the interfacial monolayer and the liposomal bilayer is graphically depicted in Figure 4. This equilibrium state is referred to as “monolayer–bilayer equilibrium” or MBE in the following. This equilibrium has been studied in biophysical models of LD biogenesis in the endothelial reticulum both empirically and theoretically [13,37].

From the measured minimum interfacial tension *γ*_min_ of the monolayer, the monolayer surface pressure *∏*_M_ can be calculated using the interfacial tension *γ*_0_ between the pure bulk phases water and PFP:

(2) $$\Pi_{M}=\gamma_{0}-\gamma_{\min}$$

This monolayer surface pressure can be transformed experimentally into the bilayer surface pressure *∏*_B_ by the Langmuir–Blodgett and the Langmuir–Schaefer technique, achieving stable bilayers in the surface pressure range of 28–42 mN/m [38]. As is shown in Table 2, the monolayer surface pressures *∏*_M_ are realistic in all cases for the corresponding surface pressures *∏*_B_ of the neighboring liposomes. This realistic range of surface pressures provides credibility to the notion that thermodynamic equilibrium is in fact reached and that the monolayer–bilayer equilibrium is established. Our results differ from those reported by Chorlay et al. who found that in the case of droplets of triglyceride incorporated in giant unilamellar vesicles, thus forming local monolayers directly connected to the PL bilayer membrane, the surface pressure *∏*_M_ is reduced by about 10% compared to ∏_B_ as measured by the micropipette aspiration technique [13]. They postulate that this discrepancy is due to the monolayer–triglyceride interaction. This comparison indicates that using a perfluorocarbon as hydrophobic (omniphobic) phase could lead to a better equivalence of *∏*_M_ and *∏*_B_—which would need to be confirmed using the micropipette aspiration technique.

Since the monolayer must be in direct contact with the coalescing liposomal bilayer during PL transfer, bilayer surface pressures must also equally be around 41 mN/m for T > T_m_ for both DPPC and DSPC. Below T_m_, the surface pressure of monolayers in equilibrium with bilayers is around 31.5 mN/m, while above T_m_ it was measured to be 45.4 mN/m for DPPC at the air/water interface [39,40]. Given the difference in the phase boundaries (air/water vs. water/PFC), the surface pressures of monolayers in equilibrium with bilayers are in very good agreement above T_m_. However, Mansour et al. 2007 could not detect any monolayer formation from coalescing liposomal bilayers at air/water interfaces below T_m_ and assumed that this was due to the state of the PL as a gel phase. We have shown that this is not true, but must be a misinterpretation caused by two artefacts: too-low PL concentrations in the aqueous phase and an insufficiently characterized size distribution of liposomes that might have had stability and sedimentation problems.

Lee at al., who investigated air/water surface tensions, describe the phenomenon that cholesterol alone does not change the surface tension (of water) but contrary to our results, cholesterol added to pure lipids leads to the same surface tension as the pure lipid. While this is in accordance with the interfacial tension reached below T_m,_ it does not apply to the results above T_m_ where the addition of cholesterol and the temperature increase does not lead to lower interfacial tensions. Thus, for PFP, cholesterol plays a minor role in changing the interfacial tension even with an increase in temperature.

The increased PL interaction of pure lipids at the interface is also mainly reflected in the calculated high Gibbs adsorption isotherms and thus in the very small area per molecule. The results are listed in Table 3. The area per molecule is calculated from the slope according to Equations (3) and (4) with

(3) $$\Gamma =-\frac{c_{0}}{\mathrm{RT}}{\left(\frac{\partial \gamma }{\partial c_{0}}\right)}_{p,T}=-\frac{1}{\mathrm{RT}}{\left(\frac{\partial \gamma }{\partial \ln c_{0}}\right)}_{p,T}$$

and

(4) $$A_{\min}=\frac{1}{\Gamma N_{A}},$$

where *Γ* is the interfacial concentration that is used to calculate the Gibbs adsorption isotherm, *c*_0_ is the aqueous concentration of PLs, *R* is the gas constant and *T* the absolute temperature. The interfacial concentration allows the calculation of the minimum area per molecule *A*_min_ by using the Avogadro constant N_A_. The area per molecule decreases to about 5 Å^2^ for both PLs above T_m_. Calculations by Li et al., who calculated the area per molecule of DPPC on chloroform, show an area of 61 Å^2^ for DPPC [41]. Hildebrandt et al. report an area of 41 Å^2^, a value that is also very different from the results presented here [42]. The discrepancy is probably due to the inappropriate calculation method for this case. The Gibbs adsorption isotherm assumes an ideal diluted solution. However, the present saturation concentration of lipids at the interface does not meet this criterion which leads to unrealistic areas per molecule. It cannot be excluded that the PLs are present in double or triple lipid layer at the interface [43,44]. An alternative computational method is presented by Li, Miller, and Möhwald [41]. Instead of the concentration-dependent calculation of the minimum surface area, they use a time-dependent approach from reaching quasi-equilibrium. For this purpose, the equilibrium interfacial tension is extrapolated towards infinity and plotted for one concentration. In the following section, a consideration of this calculation method for the determination of the area per molecule and the comparison with the concentration-dependent calculation will be presented.

### 2.2. Concentration and Time-Dependent Calculation of the Area per Molecule

**Figure 5:**
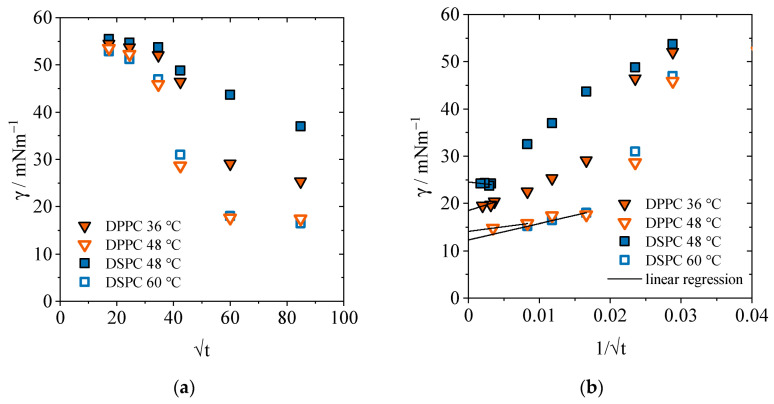
(**a**) Plot of interfacial tension *γ(t)* versus $\sqrt{t}$ to determine the diffusion coefficient *D* of the PLs DPPC and DSPC below and above the transition temperature at a concentration of 0.1 mM. (**b**) plot of interfacial tension γ(*t*) vs. $1/\sqrt{t}$ to determine the equilibrium interfacial tension *γ*_e,0.1 mM_ by extrapolation for the PLs DPPC and DSCP below and above the transition temperature, respectively, at a concentration of 0.1 mM each.

Basically, the area per molecule *A*_min_ is determined from the interfacial concentration Γ (cf. Equation (4)). This, in turn, can be calculated in different ways. In the previous section, the interfacial concentration was calculated from the slope of an interfacial tension-concentration diagram using Equation (3). The method is based on ideal diluted solutions and the assumption of a thermodynamic equilibrium which can be questioned for our case of saturation concentration and quasi-equilibrium. The strong slope leads to particularly small areas per molecule. Alternatively, the interfacial concentration can be determined using a time-dependent approach. Li, Miller, and Möhwald follow this approach in calculating the area per molecule and extrapolate the equilibrium interfacial tension [41]. For this purpose, a time-dependent measurement of the interfacial tension at a given concentration *c* is used. As a result of the time-dependent calculation method, the diffusion coefficient *D* is obtained as a characteristic quantity for the adsorption process of phospholipids. According to the diagram in Figure 5a, the measured interfacial tension *γ* is plotted against $\sqrt{t}$. For the PLs DPPC and DSPC, the adsorption process is shown below and above the respective transition temperature and at a concentration of 0.1 mM. The figures for the mixtures with cholesterol can be found in the Supplementary Material (Figure S2). The diffusion coefficient is calculated from the slope dγ/d√t, which describes the initial decrease in interfacial tension. Mathematically, the diffusion-controlled adsorption mechanism according to Ward and Tordai is given by

(5) $$\left[\frac{d\gamma }{d\surd t}\right]=\;-2\mathrm{RT}c_{0}\sqrt{\frac{D}{\pi }},$$

with *R* as the universal gas constant, *T* as the absolute temperature, and *c*_0_ as the surfactant concentration [45]. Rearranging the equation yields in the diffusion coefficient for the respective phospholipid at the corresponding temperature. The numerical values are listed in Table 4. For the determination of the interfacial concentration *Γ* by the time-dependent method, it is necessary to reach the equilibrium state. For this purpose, the interfacial tension γ is plotted versus $1/\sqrt{t}$ (Figure 5b). The Hunsel–Joos equation mathematically describes the extrapolation of *t*→∞ for the determination of the interfacial concentration with

(6) $${\left[\frac{d\gamma }{d(1/\surd t)}\right]}_{t\to \infty }=\frac{\mathrm{RT}\Gamma^{2}}{c_{0}}\sqrt{\frac{\pi }{4D}}$$

and is shown in Figure 5b [46]. The equilibrium interfacial tension is determined for the PLs DPPC and DSPC below and above the transition temperature and at a concentration of 0.1 mM. The slope $d\gamma /d(1/\sqrt{t})$ is calculated from the linear fit of the lowest measured interfacial tensions extrapolated towards infinity. From Equation (5), the calculated interfacial concentration *Γ* and the Avogadro constant N_A_ can be used to determine the minimum area per molecule of *A*_min_ given by Equation (4). The calculation of the interfacial concentration via the time-dependent method has the advantage that fluctuations due to the adsorption process can be excluded. Since the adsorption process occurs simultaneously with the formation of the interface, it cannot be resolved with sufficient accuracy because of the delayed start of the measurement process. Therefore, the comparison of both methods shows that via the concentration-dependent calculation method a clearly underestimated area per molecule is calculated, while the extrapolation is a basis for both DPPC and DSPC output areas that can be compared with literature values.

**Figure 6:**
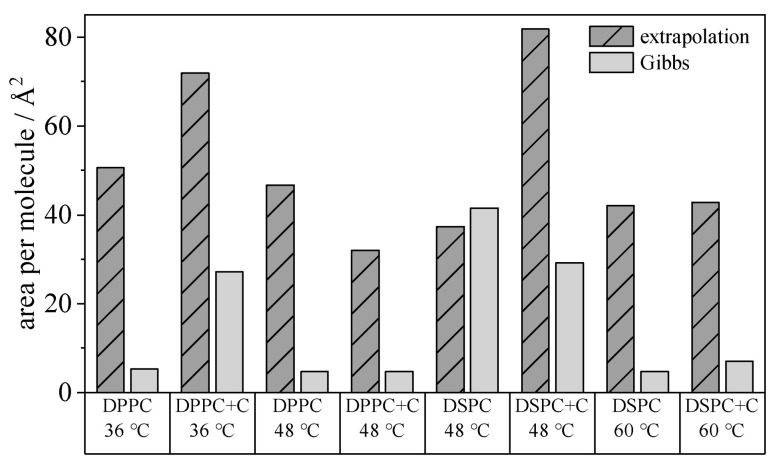
Comparison of the area per molecule *A*_min_ for the PLs DPPC, DPPC+C, DSPC and DSPC+C below and above the transition temperature, respectively. Shown as dashed bars is the approach via extrapolation of the equilibrium interfacial tension *t* → ∞ (Equation (6)) to determine the isotherms and the area calculated according to Li, Miller, and Möhwald [41]. Plotted in light grey is the calculation of the isotherms via the slope when the interfacial tension is applied as a function of concentration (Equation (3)).

The calculated areas per molecule using time- and concentration-dependent methods for the PLs DPPC, DPPC+C, DSPC and DSPC+C are shown in Figure 6. For each PL, the calculation took place below and above the transition temperature. Striped bars show the diffusion-based method (Equation (6)) to. The resulting calculated area per DPPC molecule is 50.6 and 46.7 Å^2^ (at 36 and 48 °C, respectively); for DSPC, the minimum area is 37.3 and 42.1 Å^2^ (48 and 60 °C, respectively). With cholesterol added to DPPC, *A*_min_ is 72 Å^2^ below and 31.1 Å^2^ above T_m_. These values are in broad agreement with the experimental data of Leekumjorn and Sum (65 Å^2^ for DPPC) [47]. Demel et al. describe a condensing effect of cholesterol on the determined area per molecule which can be observed here as well, but only for the temperature above T_m_ [48]. The agreement of both methods for the calculated area of DSPC at 48 °C is surprising. It is reasonable to assume that the temperature increase does not completely compensate for the steric hindrance caused by the fatty acid chains. The adsorption process is therefore slow enough to resolve this metrologically in the concentration-dependent calculation method and thus does not lead to an underestimation of the calculated area. The overall differences of *A*_min_ to literature data are due to monolayer fusion resistance at the interface for T < T_m_.

Furthermore, the data comparison in Table 4 shows that the extrapolated equilibrium interfacial tension agrees with the measured minimum interfacial tension within the standard deviations. Thus, the assumption of the minimum area per molecule extrapolated from the γ vs. $1/\sqrt{t}$ graph is consistent with our empirical data. To classify the diffusion coefficient, which was initially calculated using Equation (5), the Stokes–Einstein translational diffusion coefficient is calculated below for comparison. The calculation is shown here using DPPC as an example. The underlying hydrodynamic diameter of the DPPC molecule of *R_h_* = 17 Å is taken from the publication by Hildebrandt et al. [49]. The diffusion coefficient is calculated according to

(7) $$D^{\mathrm{SE}}(T\;)=\frac{k_{b}\;T}{6\pi \eta_{\mathrm{cont}.}R_{h}},$$

with *k_b_* as the Boltzmann constant, *T* the respective temperature and *η*_cont._ the dynamic viscosity of the continuous phase (water). This gives the following diffusion coefficients for DPPC at different temperatures: *D^SE^*(36 °C) = 2.06 × 10^−10^ m^2^/s, *D^SE^*(48 °C) = 2.51 × 10^−10^ m^2^/s, and *D^SE^*(60 °C) = 3.08 × 10^−10^ m^2^/s. The experimentally determined coefficients are much larger, indicating a slower diffusion process. Calculating the hydrodynamic radius from the experimentally determined diffusion coefficient, it becomes obvious that the PLs are not molecularly dissolved as monomers, but rather liposomes coalesce with the interface as sketched in Figure 4, with *R_h_* differing from 100–5000 nm. The diffusion coefficient becomes larger at elevated temperature. The chain length itself leads to a larger diffusion coefficient with a shorter fatty acid chain, so that despite the significantly lower steric hindrance at high temperature, this cannot be completely ruled out.

On the basis of the available data, the method according to Li, Miller and Möhwald should therefore be preferred for the determination of the area per molecule [41]. This applies in particular if the PLs have co-existences of two different states in the concentration range which is to be used for the determination according to Gibbs. The time-dependent calculation approach is also a better alternative for too-fast accumulation processes which cannot be resolved with corresponding accuracy.

## 3. Conclusions

We examined the influence of the temperature below and above T_m_ on the interfacial tension between the perfluorocarbon PFP and water with an aqueous liposomal dispersion of DPPC and DSPC as well as a mixture of those PLs and cholesterol. The area per molecule was determined by Gibbsian approach which showed very small molecular areas in comparison to the literature. It was found that due to the saturation concentration of PLs in the aqueous phase and only quasi-equilibrium instead of thermodynamic equilibrium, the calculation method does not reflect the molecular area appropriately. Instead, the time-dependent determination of the area at low concentrations and based on the diffusion of PLs to the interface leads to results that are closer to those of the literature.

In addition, it is noticeable that above T_m_, the average minimum interfacial tension does not differ between DPPC or DSPC; the same applies to the mixtures with cholesterol. This finding indicates that regardless of the chain length, the higher temperature leads to a thermodynamic equilibrium between the monolayer and liposomes. Thus, equivalent interfacial tensions occur for different PLs, as proven for DPPC and DSPC and confirmed with a t-test. In contrast to earlier results, we could show that liposomal PL bilayers also form monolayers in thermodynamic equilibrium in the range of about 30 mN/m even below T_m_, thus in the gel state.

The addition of cholesterol does not lower the interfacial tension as much as pure PLs, not even above T_m_. Hence, the major influence on the water/PFC interface comes from the PLs. Cholesterol mainly stabilizes the liposomal bilayer conformations in the aqueous phase which leads to a slower bilayer unfolding and monolayer adsorption process. This finding is confirmed by the determined diffusion coefficient. It provides results that indicate a diffusion and adsorption process of liposomes at the interface instead of monomers.

## 4. Materials and Methods

### 4.1. Materials

PLs were provided by Lipoid (Ludwigshafen, Germany). The synthetic lipids 1,2-dipalmitoyl-sn-glycero-3-phosphatidylcholine (DPPC, number of carbon atoms: degree of unsaturation = 16:0), 1,2-distearoyl-sn-glycero-3-phosphatidylcholine (DSPC, 18:0) were received in powder form and dissolved in ethanol (Carl Roth GmbH, Karlsruhe, Germany; 99%) for preparation of stock suspensions. Cholesterol was purchased from Carl Roth GmbH (Karlsruhe, Germany). The PFC **p**er**f**luoroperhydro**p**henanthrene (PFP) was acquired from F2 Chemicals (Preston, UK).

### 4.2. Preparation of Lipid Suspensions

For a detailed description of the preparation of lipid suspensions, please refer to Ullmann et al. [31]. In short, stock suspensions of 20 mM of each lipid were prepared with the film hydration method in ethanol and rehydrated in double distilled water. Ultrasound with a 100% cycle and 10% amplitude (Digital Sonifier 450, Branson Ultrasonic Corporation, Danbury, CT, USA) was applied for 10 s followed by a 50% cycle and 10% amplitude for 10 min for a better dispersion of lipids, followed by extrusion through track-etched membranes. The final concentration of suspensions was determined via phosphorus assay according to Fiske [50]. Stock suspensions were brought to the respective temperature and diluted to the desired concentration. Between experiments, they were stored in a fridge at 4 °C. For mixtures, cholesterol was added to the PL in a molar ratio of 60:40 (phospholipid:cholesterol; DPPC+C and DSPC+C).

### 4.3. Tensiometry

A previous investigation discussed the difficulty of determining the interfacial tension between PFP and an aqueous phase containing PL suspensions [31]. It proved that the Du Noüy Ring is equally precise for interfacial measurements between two immiscible phases as the spinning drop method, but much more versatile in long-term and parellelized tensiometric studies. Hence, the Du Noüy Ring tensiometer (DCAT E11, DataPhysics, Filderstadt, Germany) was applied in the following experiments. The tensiometer was placed in a temperature-controlled room (20.0 ± 0.1 °C) and samples were kept at the investigated temperature by using a thermostatic barrel with a water bath. Liquids were preheated to the respective temperature and glass vessels with oil and PL suspension were stored in a heating chamber (Memmert, Schwabach, Germany) between concentration-dependent long-term measurements. The glass vessels were sealed with parafilm^®^ to avoid evaporation of the aqueous PL suspension. Prior to the concentration-dependent change of interfacial tensions *γ*, quasi-equilibrium *γ_ε_* was examined for each PL (DPPC, DSPC) or mixture (DPPC+C, DSPC+C) at a concentration of 0.1 mM. The time *t_ε_*, after which *γ_ε_* is reached, was defined as the empirical value for which the condition: *∆γ/∆t* < 0.1 mN/m per hour holds. Interfacial tensions at different concentrations for the PLs and mixtures were then investigated at different temperatures, where each data point comprised the quasi-equilibrium *γ_ε_* at *t_ε_*. Each experiment was carried out in triplicate. For a detailed description of interfacial measurements, please refer to [31].

The so-called main transition temperature T_m_ of the pure PLs and the studied temperatures are listed in Table 5. A temperature of 48 °C was chosen to compare two exact temperatures of both lipids with each other. For DPPC, that temperature is 7 °C above T_m_; for DSPC, it is 7 °C below T_m_. In addition, a second temperature 5 °C below and above the respective T_m_ was studied. The chosen temperatures are close to T_m_, but not too close to show no effect. Optionally, cholesterol was added to DPPC and DSPC and measured at the same temperatures. The literature reports that for those mixtures, there is no main transition at molar fractions >0.45 for DPPC and >0.5 for DSPC because only a liquid state exists beyond those concentrations [34,36]. From the transition temperature, it can already be hypothesized that the different chain lengths result in a temperature dependence of the interfacial tension. It was necessary to run time-dependent measurements to determine the quasi-equilibrium of each lipid at each selected temperature in order to subsequently determine the interfacial tension in a concentration-dependent manner.

## Figures and Tables

**Table 1:** Average quasi-equilibrium times *t_ε_* determined at *Δγ/Δt* < 0.1 mN/m for the PLs DPPC, DPPC+C and DSPC at the respective temperatures.

Temperature/°C	*t_ε_*_,DPPC_/h	*t_ε_*_,DPPC+C_/h	*t_ε_*_,DSPC_/h	*t_ε_*_,DSPC+C_/h
20	<48 ^1^	<48	<168 ^1^	<168
36	<48	<48	-	-
48	<24	<48	<72	<168
60	-	-	<24	<144

^1^ Data from Ullmann et al. [31].

**Table 2:** Summarized values for the two-sided t-test for independent samples with a level of significance of α = 0.05 and for the hypothesis H_0_ that the average quasi-equilibrium interfacial tensions ${\overset{-}{\gamma }}_{\varepsilon }$ above T_m_ for both lipids and for *c* > 0.1 mM equal each other.

Hypothesis H_0_	df	Critical *t*-Value	Calculated *t*-Value	*p*-Value
${\overset{-}{\gamma }}_{\varepsilon }$(DPPC) = ${\overset{-}{\gamma }}_{\varepsilon }$(DSPC)	6	2.36	−2.07	0.079
${\overset{-}{\gamma }}_{\varepsilon }$(DPPC+C) = ${\overset{-}{\gamma }}_{\varepsilon }$(DSPC+C)	7	2.44	0.14	0.89

**Table 3:** Comparison of the MBE in mM and the minimum area *A*_min_ per molecule in Å^2^ as well as the minimum measured interfacial tension *γ*_min_ for the investigated PLs DPPC, DPPC+C and DSPC, respectively below and above the transition temperature T_m_. Monolayer surface pressure *∏*_M_ for each.

Phospholipid (Temperature)	MBE/mM	*A*_min_/Å^2^ [Equation (3)]	$\gamma_{\min}\;\pm \;\bar{s}$/mN m^−1^	*∏*_M_/mN/m
DPPC (36 °C)	0.100	5.3	19.31 ± 0.93	35.69
DPPC (48 °C)	0.101	4.7	13.67 ± 0.45	41.33
DPPC+C (36 °C)	0.530	27.1	26.35 ± 1.81	28.65
DPPC+C (48 °C)	0.101	4.8	24.28 ± 3.73	30.72
DSPC (48 °C)	0.101	41.6	24.16 ± 1.47	30.84
DSPC (60 °C)	0.103	4.7	14.34 ± 1.34	40.66
DSPC+C (48 °C)	0.934	29.2	25.33 ± 1.62	29.67
DSPC+C (60 °C)	0.110	7.0	23.28 ± 4.34	31.72

**Table 4:** Tabular comparison of the diffusion coefficient *D* from extrapolation and the calculated minimum area per molecule of *A*_min,E_ from extrapolation and *A*_min,Gibbs_ from the concentration-dependent calculation method. Comparison of the extrapolated equilibrium interfacial tension *γ*_e,0.1 mM_ (*t* → ∞) for the PLs DPPC and DSPC and their mixtures with cholesterol at a concentration of 0.1 mM below and above the transition temperature, respectively, the quasi-equilibrium value *γ*_ε,0.1 mM_ at 0.1 mM from empirical data, and the minimum interfacial tension *γ*_min,1 mM_ at 1 mM from the experimental data.

Phospholipid (Temperature)	*D*/ m^2^ s^−1^	*A*_min, E_ /Å^2^	*A*_min,G_ /Å^2^	$\gamma_{e,0.1mM}$ (t→∞)$\;\pm \;\bar{s}$ /mN m^−1^	$\gamma_{\varepsilon ,0.1mM}$$\;\pm \;\bar{s}$/mN m^−1^	$\gamma_{min,1mM}$$\;\pm \;\bar{s}$/mN m^−1^
DPPC (36 °C)	2.43 × 10^−13^	50.6	5.3	18.50 ± 0.72	19.05 ± 0.50	19.31 ± 0.93
DPPC (48 °C)	2.30 × 10^−12^	46.7	4.7	14.11 ± 0.00	14.81 ± 0.33	13.67 ± 0.45
DPPC+C (36 °C)	7.51× 10^−14^	71.9	27.1	51.44 ± 0.13	51.44 ± 1.00	26.35 ± 1.81
DPPC+C (48 °C)	7.70× 10^−14^	32.1	4.8	21.74 ± 1.69	28.28 ± 0.95	24.28 ± 3.73
DSPC (48 °C)	1.14 × 10^−13^	37.4	41.6	24.53 ± 0.60	24.19 ± 1.08	24.16 ± 1.47
DSPC (60 °C)	1.23 × 10^−12^	42.1	4.7	12.31 ± 0.10	18.40 ± 1.40	14.34 ± 1.34
DSPC+C (48 °C)	8.30 × 10^−14^	81.8	29.2	51.26± 0.17	52.16 ± 0.64	25.33 ± 1.62
DSPC+C (60 °C)	1.57 × 10^−15^	42.9	7.0	16.27 ± 0.82	28.72 ± 0.86	23.28 ± 4.34

**Table 5:** Main transition temperature T_m_ of each PL as well as the examined temperatures T_1_ below (T_1_ < T_m_) and above (T_2_ > T_m_) the transition temperatures of DPPC and DSPC.

Phospholipid	T_m_/°C	T_1_ < T_m_/°C	T_2_ > T_m_/°C
DPPC	41	36	48
DSPC	55	48	60

## Data Availability

Not applicable.

## Supplementary Materials

The following supporting information can be downloaded at: https://www.mdpi.com/article/10.3390/gels9100803/s1, Figure S1: Interfacial tension γ as a function of time t in dependence of the temperature for the pure phospholipid DSPC and in a mixture with cholesterol (DSPC+C = 60:40 mol-%) at a concentration of 0.1 mM. (a) Evolution towards the quasi-equilibrium γε below the transition temperature of DSPC. (b): Evolution towards the quasi-equilibrium γε above the transition temperature of DSPC; Figure S2:(a) Plot of interfacial tension γ(t) versus √t to determine the diffusion coefficient of the mixtures DPPC+C and DSPC+C below and above the transition temperature at a concentration of 0.1 mM. (b) plot of interfacial tension γ(t) vs. 1/√t to determine the equilibrium interfacial tension by extrapolation for the mixture DPPC+C and DSCP+C below and above the transition temperature, respectively, at a concentration of 0.1 mM each.
