# Supplementary material for: Monolayer/Bilayer Equilibrium of Phospholipids in Gel or Liquid States: Interfacial Adsorption via Monomer or Liposome Diffusion?
Source: Gels. 2023 Oct 6;9(10):803. doi: 10.3390/gels9100803 (PMC10606027; doi:10.3390/gels9100803)
Supplement: Supplementary file 1 [file gels-09-00803-s001.zip › Supplementary Material Ullmann et al.pdf]

Supplementary Material

Monolayer/Bilayer equilibrium of Phospholipids in gel or liquid states: Interfacial Adsorption via Monomer or Liposome diffusion?

Kirsten Ullmann [1,*], Lea Fachet [1], Hermann Nirschl [1] and Gero Leneweit [2,*]

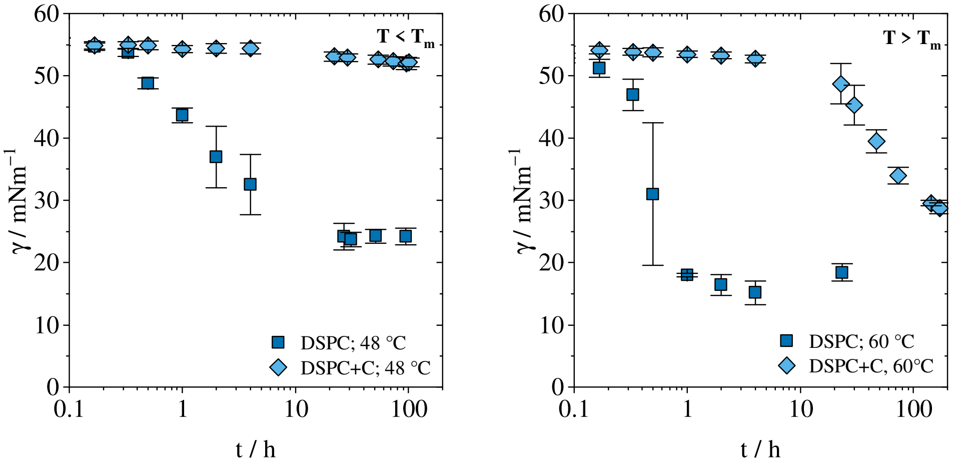

(a) (b)

Figure S1: Interfacial tension γ as a function of time t in dependence of the temperature for the pure phospholipid DSPC and in a mixture with cholesterol (DSPC+C = 60:40 mol-%) at a concentration of 0.1 mM. (a) Evolution towards the quasi-equilibrium γ_ε below the transition temperature of DSPC. (b): Evolution towards the quasi-equilibrium γ_ε above the transition temperature of DSPC.

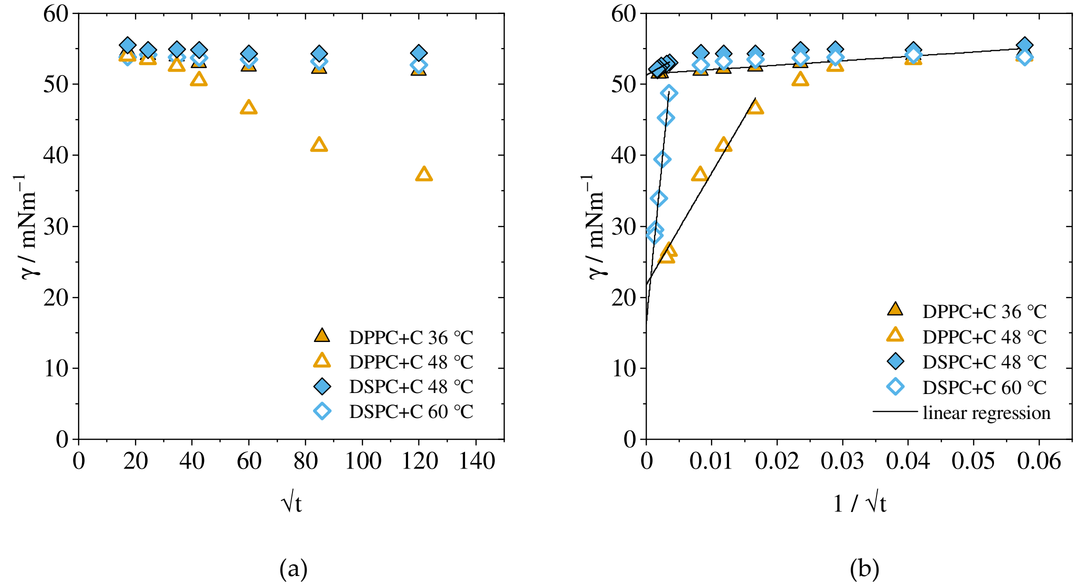

Figure S2: (a) Plot of interfacial tension γ(t) versus $\sqrt{t}$ to determine the diffusion coefficient of the mixtures DPPC+C and DSPC+C below and above the transition temperature at a concentration of 0.1 mM. (b) plot of interfacial tension γ(t) vs. $1/\sqrt{t}$ to determine the equilibrium interfacial tension by extrapolation for the mixture DPPC+C and DSCP+C below and above the transition temperature, respectively, at a concentration of 0.1 mM each.
